# Cisplatin-based concurrent chemoradiotherapy improved the survival of locoregionally advanced nasopharyngeal carcinoma after induction chemotherapy by reducing early treatment failure

**DOI:** 10.1186/s12885-022-10237-8

**Published:** 2022-11-29

**Authors:** Xing-Li Yang, Lu-Lu Zhang, Jia Kou, Guan-Qun Zhou, Chen-Fei Wu, Ying Sun, Li Lin

**Affiliations:** 1 Department of Radiation Oncology, The First Affiliated Hospital, Sun Yat-sen University, 510060 Guangzhou, People’s Republic of China; 2 Department of Molecular Diagnostics, State Key Laboratory of Oncology in South China, Guangdong Key Laboratory of Nasopharyngeal Carcinoma Diagnosis and Therapy, Collaborative Innovation Center for Cancer Medicine, Sun Yat-sen University Cancer Center, 510060 Guangzhou, People’s Republic of China; 3 Department of Anaesthesiology, State Key Laboratory of Oncology in South China, Guangdong Key Laboratory of Nasopharyngeal Carcinoma Diagnosis and Therapy, Collaborative Innovation Center for Cancer Medicine, Sun Yat-sen University Cancer Center, 510060 Guangzhou, People’s Republic of China; 4 Department of Radiation Oncology, State Key Laboratory of Oncology in South China, Guangdong Key Laboratory of Nasopharyngeal Carcinoma Diagnosis and Therapy, Collaborative Innovation Centre of Cancer Medicine, Sun Yat-sen University Cancer Centre, 651 Dongfeng Road East, 510060 Guangzhou, People’s Republic of China

**Keywords:** Nasopharyngeal carcinoma, Chemotherapy, Cisplatin dose, Failure, Survival

## Abstract

**Purpose:**

The aims of this study focusing on Locoregionally advanced nasopharyngeal carcinoma (LANPC) were mainly two-fold: on the one hand, to establish a cut-off value to differentiate early and late failure based on prognosis after recurrence or metastasis; and on the other hand, to investigate the duration of concurrent cisplatin benefit over follow-up time. The results of our study have the potential to guide clinical practice and follow-up.

**Methods:**

In total, 3123 patients with stage III-IVa NPC receiving Induction chemotherapy followed by concurrent cisplatin or not were analysed. The cut-off value of treatment failure was calculated using the minimum *P*-value approach. Random survival forest (RSF) model was to simulate the cumulative probabilities of treatment failure (locoregional recurrence and /or distant metastasis) over-time, as well as the monthly time-specific, event-occurring probabilities, for patients at different treatment groups.

**Results:**

Based on subsequent prognosis, early locoregional failure (ELRF) should be defined as recurrence within 14 months (*P* = 1.47 × 10 − 3), and early distant failure (EDF) should be defined as recurrence within 20 months (*P* = 1.95 × 10 − 4). A cumulative cisplatin dose (CCD) > 200 mg/m^2^ independently reduced the risk of EDF (hazard ratio, 0.351; 95% confidence interval (CI), 0.169–0.732; *P* = 0.005). Better failure-free survival (FFS) and overall survival (OS) were observed in concurrent chemotherapy settings ([0 mg/m^2^ vs. 1-200 mg/m^2^ vs. >200 mg/m^2^]: FFS: 70.4% vs. 74.4% vs. 82.6%, all *P* < 0.03; OS: 79.5% vs. 83.8% vs. 90.8%, all *P* < 0.01). In the monthly analysis, treatment failure mainly occurred during the first 4 years, and the risk of distant failure in patients treated with concurrent chemotherapy never exceeded that of patients without concurrent chemotherapy.

**Conclusion:**

Locoregional failure that developed within 14 months and/or distant failure within 20 months had poorer subsequent survival. Concurrent chemotherapy provides a significant FFS benefit, primarily by reducing EDF, translating into a long-term OS benefit.

**Supplementary Information:**

The online version contains supplementary material available at 10.1186/s12885-022-10237-8.

## Background

Nasopharyngeal carcinoma (NPC) is considered a special type of head and neck cancer due to its unique epidemiology, skewed pathology and preeminent response to treatment. In 2018, there were 129,079 new cases and 72,987 deaths worldwide [1]. Nearly 80% of patients develop locally advanced disease and are at high risk of recurrence, as patients at an early stage present anonymous symptoms in most cases [2, 3].

Considering of complex anatomical location and high radio- and chemo-sensitivity, platinum-based chemoradiotherapy has been the main treatment modality for non-disseminated NPC [4]. Data from large-scale multi-centre phase II-III randomized controlled trials showed promising results by adding induction chemotherapy (IC) before concurrent chemoradiotherapy (CCRT) in locoregionally advanced NPC (LANPC) [5–7]. IC plus CCRT was recommended for LANPC in the latest National Comprehensive Cancer Network (NCCN) Guidelines.

Cisplatin administered during radiotherapy is supposed to further increase radiosensitivity and eliminate micrometastasis after IC. For such cases, the cumulative cisplatin dose (CCD) in concurrent chemotherapy plays an important role in conferring survival benefits. Standard practice for concurrent chemotherapy dosage included cisplatin-based regimens delivered either weekly (40 mg/m^2^) for 7 cycles or triweekly (100 mg/m^2^ or at least 80 mg/m^2^) for 3 cycles [4, 8, 9]. Nevertheless, a significant fraction of patients failed to finfish the last cycles of cisplatin due to the accumulation of toxicity of radiotherapy and chemotherapy [10]. Although objectives and treatment protocols vary, 200 mg/m^2^ is the optimal cut-off value in most studies [8, 9, 11–13] and is recommended in the joint guidelines of the CSCO and ASCO [4].

Kaplan-Meier plots, typically used to present the recurrence or survival data from clinical trials, convey information regarding the absolute rate of survival at any given time point. However, a separation in survival curves is unable to convey a continuing difference in the risk of an event because an early advantage is maintained over time in such presentations. Whether concurrent cisplatin cures disease, provides continued survival benefit or temporarily inhibits tumours, thus postponing disease failure, remains unknown.

The timing of treatment failure is supposed to be significant for overall survival, and early recurrence is more likely to induce lower survival than late recurrence, which may imply tumour biology and treatment effects. However, the cut-off values of “early recurrence’’ varied arbitrarily from 6 to 24 months in different studies [14–18]. Based on prognosis after recurrence, pancreatic ductal adenocarcinoma, gastric cancer and rectal cancer established the optimal definition of “early recurrence” within 12 months [19–21]. In NPC patients, Li et al. confirmed the negative survival impact and summarized the characteristics of early recurrence with a threshold of 2 years based on previous studies. They also found that post-recurrence treatment options (*P* = 0.000) independently indicated post-recurrence OS [16]. To date, a prognostic evidence-based cut-off value for NPC is lacking.

## Methods

The aims of this study focusing on LANPC were mainly two-fold: on the one hand, to establish a cut-off value to differentiate early and late failure based on prognosis after recurrence or metastasis; and on the other hand, to investigate the duration of concurrent cisplatin benefit over follow-up time. The results of our study have the potential to guide clinical practice and follow-up.

### Patient selection

From April 2009 to December 2015, a total of 10,126 consecutive patients with newly diagnosed, histologically proven, non-disseminated NPC in Sun Yat-sen University Cancer Center (SYSUCC) were reviewed using an NPC-specific database of the big-data intelligence platform. All patients were restaged according to the 8th edition of the American Joint Committee on Cancer/International Union against Cancer (AJCC/UICC) staging system based on MRI imaging and medical records. Finally, we identified 3123 patients in this study with the following inclusion criteria: (a) III–IVa disease (according to the 8th AJCC/UICC staging system); (b) induction chemotherapy and radical intensity-modulated radiation therapy (IMRT) ± cisplatin-based concurrent chemotherapy; and (c) no other malignancies or severe heart, lung, liver, and kidney diseases.

### Treatment and study endpoints

Cisplatin-based concurrent chemotherapy was administered triweekly for one to three cycles (80–100 mg/m^2^) or weekly (30–40 mg/m^2^) for a maximum of 7 cycles at the initiation of IMRT. IC comprised cisplatin (75 mg/m ^2^ ) with 5-fluorouracil (600 mg/m ^2^ ) and docetaxel (60 mg/m ^2^ ), cisplatin (80 mg/m ^2^ ) with docetaxel (75 mg/m ^2^ ), cisplatin (80 mg/m ^2^ ) with 5-fluorouracil (1000 mg/m ^2^ ), or gemcitabine (1000 mg/m ^2^ ) with cisplatin (80 mg/m ^2^ ) triweekly.

Overall Survival (OS, freedom from treatment initiation to death for any cause) was the primary endpoint in this study. Other endpoints were failure-free survival (FFS, freedom from treatment initiation to first event or death from any cause), distant failure-free survival (DFFS, freedom from treatment initiation to first distant metastasis), locoregional failure-free survival (LRFFS, freedom from treatment initiation to the first local or regional recurrence or both), and post-failure OS (defined as OS minus LRFFS or DFFS), respectively.

After treatment completion, patients returned to the hospital for review every 3 months during the first 2 years and every 6 months after that until death. Patients were followed up via telephone if their recent examination records were not available. Follow-up duration was defined as the date of treatment initiation until last contact or death. Posttreatment surveillance at each follow-up appointment included a comprehensive physical examination, plasma EBV DNA load, nasopharyngoscopy,and imaging assessment similar to the pretreatment examinations. Clinical suspicion of treatment failure was confirmed based on imaging assessment with or without cytological biopsies.

### Statistical analysis

All statistical analyses were implemented using R version 3.4.4 (http://www.r-project.org) and the SPSS package for Windows, version 23.0 (Chicago, IL).

A minimum *P*-value approach with correction by Bootstrap method was used to calculate the cut-off value [19, 20]. The optimal thresholds of LRFFS and DFFS to divide the patients into an early and late failure cohort were based on survival after treatment failure [22]. In this approach, the log-rank test was performed for different lengths of LRFFS and DFFS to determine the optimal cut-off point with the lowest *P* value. The R code is available in the supplementary files 1. Survival curves were estimated using the Kaplan–Meier method and differences were examined by the log-rank test.

The patients’ clinicopathological and treatment characteristics were compared using the χ2 or Fisher’s exact test. Multivariate analysis, calculated using the Cox proportional hazards models, was used to test for independent significance by backward elimination of insignificant explanatory variables. Variables with a *P* value of < 0.10 were included as covariates in the Cox proportional hazards model. The criterion for statistical significance was set at *P*-values < 0.05 based on two-sided tests.

A random survival forest (RSF) model was used to simulate the cumulative probabilities of treatment failure (locoregional recurrence and/or distant metastasis) over time, as well as the monthly event-occurring probabilities, for patients in different treatment groups. We used a forest of 1000 random bootstrap survival trees to yield a stable result from right censored survival data [23, 24]. The curves of cumulative probability represented disease failure over a period of time. Monthly specific probabilities were calculated based on the derivative of the curves. The curves were adjusted for risk factors, including sex, age, smoking status, family history, T stage, and N stage, in this study. RSF methodology was performed by the random forest SRC package (version 2.6.1) [25]. The R code referred to the study conducted by Zhou et al. [26].

## Result

### Patient characteristics and treatment failure

The clinical and treatment characteristics of the patients in this cohort are summarized in Table 1. The median age at diagnosis was 44 years (range, 18–76 years); 49.1% (1536/3123) of subjects were at stage III, and 50.8% (1587/3123) were at stage IVa. In total, 56.3% (17,658/3123) of patients received 1–2 cycles of IC, and 43.7% (1365/3123) received more than 2 cycles. TPF was the most commonly used IC regimen (43.2%; 1350/3123). For 77.3% (2414/3123) receiving concurrent cisplatin, 2230 patients received CCD ≤ 200 mg/m^2^, and only 185 patients received CCD > 200 mg/m^2^.

**Table 1:** Patient demographics and clinical characteristics

Characteristic	No. of patients			Total	*P* value
	**CCD = 0 mg/m2**	**CCD(1-200 mg/m2)**	**CCD > 200 mg/m2**		
	708(100%)	2230(100%)	185(100%)	3123(100%)	
**Sex**					0.417
Male	518(73.2%)	1663(74.6%)	144(77.8%)	2325(74.4%)	
Female	190(26.8%)	567(25.4%)	41(22.2)	798(25.6%)	
**Histology**					0.550
WHO Type I-II	22(3.1%)	63(2.8%)	3(1.6%)	87(2.8%)	
WHO Type III	687(96.9%)	2168(97.2%)	182(98.4%)	3036(97.2%)	
**Age, year**					< 0.001
≤ 45	319(45.1%)	1246 (55.9%)	129(69.7%)	1694(54.2%)	
××45	389(54.9%)	1040(44.1%)	56(30.3%)	1429(45.7%)	
**Smoking history**					0.001
No	463(65.4%)	1352(60.6%)	135(73.0%)	1950(62.4%)	
Yes	245(34.6%)	878(39.4%)	50(27.1%)	1173(37.6%)	
**Drinking history**					0.025
No	628(88.7%)	1887(84.6%)	160(86.5%)	2675(85.6%)	
Yes	80(11.3%)	343(15.4%)	25(13.5%)	448(14.4%)	
**Family history of cancer**					0.158
No	535(75.6%)	1644(73.7%)	127(68.6%)	2306(73.8%)	
Yes	173(24.4%)	586(26.3%)	58(31.4%)	817(26.2%)	
T stage^*^					0.461
T1-2	99(14.0%)	287(12.9%)	29(15.7%)	415(13.3%)	
T3-4	609(86.0%)	1943(87.1%)	156(84.3%)	2708(86.7%)	
N stage^*^					0.007
N0-1	369(52.1%)	1028(46.1%)	98(53.0%)	1495(47.9%)	
N2-3	339(47.9%)	1202(53.9%)	87(47.0%)	1628(52.1%)	
Overall stage^*^					< 0.001
III	423(59.7%)	1035 (46.4%)	78(42.2%)	1536(49.1%)	
IVa	285(40.3%)	1195(53.6%)	107(57.8%)	1587(50.8%)	
**EBV DNA load, copy/ml**					0.151
< 4000	325(45.9%)	932(41.8%)	81(43.8%)	1338(42.8%)	
≥ 4000	383(54.1%)	1298(58.2%)	104(56.2%)	1785(57.2%)	
** IC cycles**					< 0.001
≤ 2 cycles	465(65.7%)	1226(55.0%)	67(36.2%)	1758(56.3%)	
> 2 cycles	243(34.3%)	1004(45.0%)	118(63.8%)	1365(43.7%)	
**ELRF**					0.950
No	694(98.0%	2188(98.1%)	182(98.4%)	3064(98.1%)	
Yes	14(2.0%)	42(1.9%)	3(1.6%)	59(1.9%)	
**LLRF**					0.485
No	621(87.7%)	1987(89.1%)	167(90.3%)	2775(88.9%)	
Yes	87(12.3%)	243(10.9%)	18(9.7%)	348(11.1%)	
**EDF**					0.045
No	636(89.8%)	2032(91.1%)	177(95.7%)	2845(91.1%)	
Yes	72(10.2%)	198(8.9%)	8(4.3%)	278(8.9%)	
**LDF**					0.700
No	653(92.2%)	2063(92.5%)	174(94.1%)	2890(92.5%)	
Yes	55(7.8%)	167(7.5%)	11(5.9%)	233(7.5%)	

*Abbreviations:* *ELRF* Early locoregional failure, *LLRF* Late locoregional failure, *EDF* Early distant failure, *LDF* Late distant failure, *WHO* World Health Organization, *EBV* Epstein–Barr virus, *CCD* Cumulative cisplatin dose

^*^According to the 8th edition of the American Joint Commission on Cancer staging system

The median follow-up of the entire cohort was 67.5 months (interquartile range 59.5–81.5 months). During the follow-up period, 13.0% (407/3123) of patients experienced locoregional recurrence, 16.4% (511/3123) experienced distant metastasis, and 19.8% (619/3123) died. The 5-year OS, FFS, LRFFS and DFFS values of the whole cohort were 83.2%, 74.0%, 89.9% and 84.1%, respectively. Among the patients who experienced locoregional recurrence, 87 received surgery, 118 received radiotherapy, 159 received chemotherapy, and 6 received comprehensive therapy as salvage treatment. Among patients with distant metastasis, 54 patients received surgery, 34 patients received radiotherapy, 273 patients received chemotherapy, and 26 received comprehensive therapy as salvage treatment (Supplementary Table 1).

### Early or late treatment failure

**Fig. 1:**
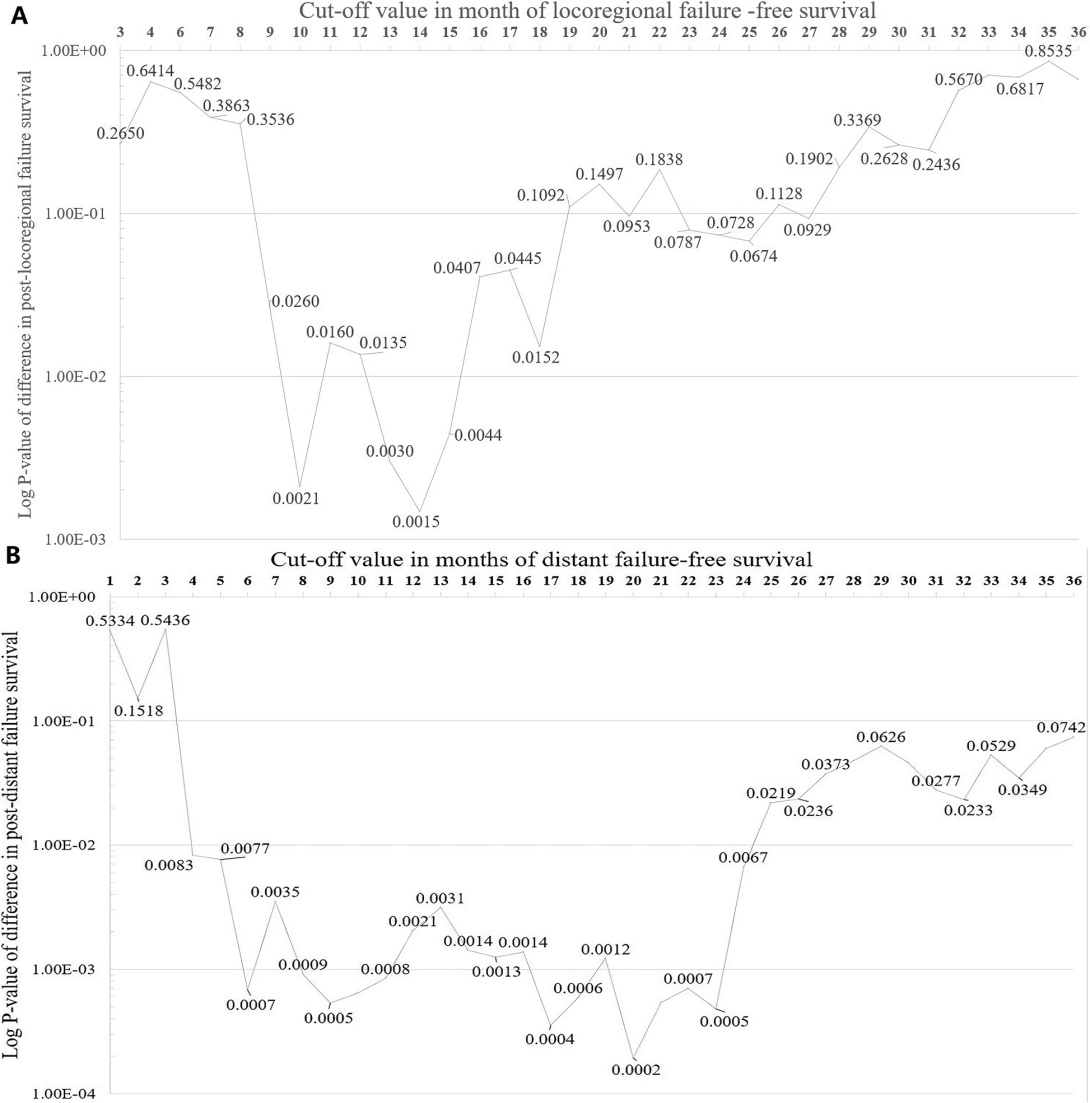
Different cut-off thresholds with corresponding *P* values for locoregional failure (**A**) and distant failure (**B**)

**Fig. 2:**
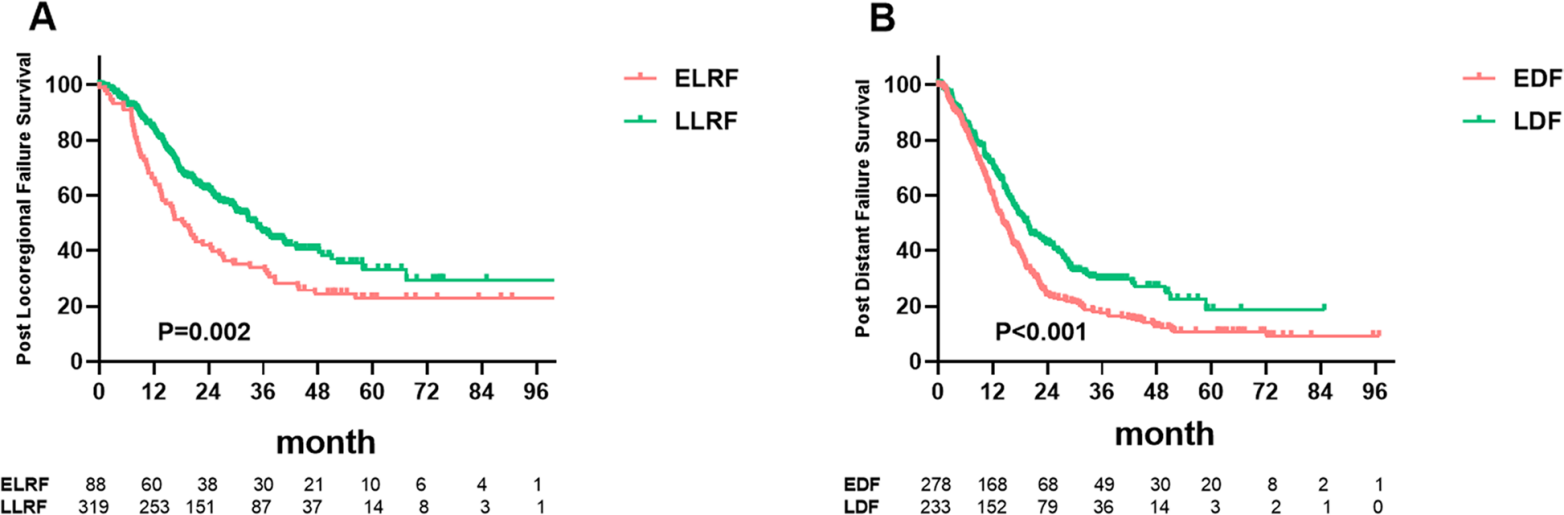
Kaplan–Meier analysis of post-failure survival in 407 patients with locoregional failure stratified by early (ELRF) and late locoregional failure (ELRF) (**A**); 511 patients with distant failure stratified by early (EDF) and late distant failure (EDF) (**B**)

The *P*-values of differences in post-failure OS regarding various cut-off points to evaluate early locoregional recurrence or distant metastasis are shown in Fig. 1. At a median follow-up of 26.9 months (interquartile range 15.7–40.0 months), the optimal length of early LRFFS (ELRFFS), based on subsequent post-recurrence survival, was 14 months (*P* = 1.47 × 10^− 3^, Fig. 1A). In the current study cohort of 407 patients with locoregional recurrence, 88 patients (21.6%) developed early locoregional failure, with 1- and 3-year post-failure OS rates of 65.9% and 34.1%, and 319 patients (78.4%) developed late locoregional failure, with 1- and 3-year post-failure OS rates of 83.8% and 46.8% (*P* = 0.002, Fig. 2A). At a median follow-up of 18.5 months (interquartile range 10.6–32.8 months), the optimal length of DFFS to distinguish between early and late distant failure was within 20 months (*P* = 1.95 × 10^− 4^, Fig. 1B). For 511 patients with distant metastasis, 278 patients (54.4%) with early metastasis had 1- and 3-year post-failure OS rates of 60.1% and 17.6%, respectively, compared with 71.3% and 29.8% for the late metastasis group (*P* < 0.001, Fig. 2B).

Multivariable analyses of locoregional failure cohort suggest ELRFFS (hazard ratio (HR), 4.606; 95% confidence interval (CI), 3.391–6.257; *P* < 0.001), post failure treatment strategy (*P* < 0.001) and clinical stage (HR, 1.639; 95% CI, 1.283–2.170; *P* = 0.001), were independently risk factors for OS (Table 2). In distance failure cohort, EDFFS (HR, 4.239; 95% CI, 3.408–5.273; *P* < 0.001) and post failure treatment strategy (*P* < 0.001) were independently risk factors for OS (Table 3).

**Table 2:** Univariate and multivariate analysis of post-failure prognostic factors in the distant failure group

	Ultivariable analysis		Multivariable analysis	
**Variable**	**HR 95%CI**	***P*** **value**	**HR 95%CI**	***P*** **value**
**Sex(Female vs. Male)**	1.170(0.910–1.503)	0.220	NA	
**WHO Histology (I ~ II vs. III)**	1.027 (0.648–1.629)	0.908	NA	
**Age, year (≤ 45vs.××45)**	0.728(0.597–0.888)	0.002	NA	
**T stage**^**a**^**( T3-4 vs. T1-2)**	1.029(0.782–1.355)	0.837	NA	
**N stage**^**a**^**( N2-3 vs. N0-1)**	1.178(0.947–1.466)	0.141	NA	
**Overall stage**^**a**^**( IVa vs. III)**	1.089(0.886–1.338)	0.418	NA	
**EBV DNA load, copy/ml** **(≥ 4000 vs. <4000)**	1.209(0.965–1.514)	0.098	NA	
**IC cycle(2cycle vs. >2 cycle)**	0.925(0.759–1.128)	0.440	NA	
**CCD, mg/m** ^**2**^		0.505	NA	
0	Reference			
1-200	0.899(0.717–1.128)	0.359		
> 200	0.748(0.411–1.361)	0.341		
**EDFFS**	1.472(1.199–1.907)	< 0.001	1.375(1.118–1.691)	0.003
**Post treatment**		< 0.001		< 0.001
**Supportive treatment**	Reference			
**Surgery**	0.128(0.084–0.197)	< 0.001	0.134(0.087–0.205)	< 0.001
**Radiotherapy**	0.291(0.191–0.444)	< 0.001	0.198(0.198–0.459)	< 0.001
**Chemotherapy**	0.255(0.201–0.325)	< 0.001	0.259(0.203–0.329)	< 0.001
**Comprehensive therapy**	0.090(0.049–0.165)	< 0.001	0.093(0.051–0.169)	< 0.001
**unknown**	0.176(0.072–0.433)	< 0.001	0.205(0.083–0.507)	0.001

*Abbreviations:* *HR* Hazard ratio, *CI* Confidence interval, *WHO* World Health Organization, *EBV* Epstein–Barr virus, *CCD* Cumulative cisplatin dose, *EDFFS* Early distant failure-free survival

^a^According to the 8th edition of the American Joint Commission on Cancer staging system

**Table 3:** Univariate and multivariate analysis of post-failure prognostic factors in the locoregional recurrence group

	ultivariable analysis		Multivariable analysis	
**Variable**	**HR 95%CI**	***P*** **value**	**HR 95%CI**	***P*** **value**
**Sex(Female vs. Male)**	0.888(0.642–1.229)	0.474		
**WHO Histology (I ~ II vs. III)**	0.729(0.902–1.614)	0.729		
**Age, year (××45 vs. ≤45)**	1.092(0.841–1.419)	0.508		
**T stage**^**a**^**( T3-4 vs. T1-2)**	1.112(0.722–1.712)	0.629		
** N stage**^**a**^**( N2-3 vs. N0-1)**	0.861(0.662–1.118)	0.262		
**Overall stage**^**a**^**( IVa vs. III)**	1.611(1.223–2.121)	< 0.001	1.544(1.166–2.044)	0.002
**EBV DNA load, copy/ml** **(≥ 4000 vs. <4000)**	1.085(0.825–1.427)	0.559		
**IC cycle(> 2 cycle vs. 2cycle)**	0.983(0.757–1.277)	0.898		
**CCD, mg/m** ^**2**^		0.746		
0	Reference			
1-200	0.954(0.703–1.293)	0.761		
> 200	0.760(0.376–1.537)	0.446		
**ELRFFS**	1.640(1.230–2.185)	0.001	1.833(1.367–2.458)	< 0.001
**Post treatment**		< 0.001		< 0.001
**Supportive treatment**	Reference			
**surgery**	0.165(0.095–0.284)	< 0.001	0.144(0.083–0.251)	< 0.001
**radiotherapy**	0.358(0.223–0.575)	< 0.001	0.360(0.224–0.578)	< 0.001
**Chemotherapy**	0.555(0.354–0.869)	0.010	0.483(0.306–0.761)	0.002
**Comprehensive treatment**	0.486(0.115–2.057)	0.327	0.554(0.131–2.348)	0.422
**Unknown**	0.391(0.053–2.896)	0.358	0.359(0.048–2.668)	0.317

*Abbreviations:* *HR* Hazard ratio, *CI* Confidence interval, *WHO* World Health Organization, *EBV* Epstein–Barr virus, *CCD* Cumulative cisplatin dose, *ELRFFS* Early locoregional failure- free survival

^a^According to the 8th edition of the American Joint Commission on Cancer staging system

Table 4 illustrates the multivariate analysis results of the whole cohort. The WHO histological type and overall stage were independently associated with LRFFS, in which the WHO histological type was an independent risk factor for early LRFFS (hazard ratio (HR), 0.354; 95% confidence interval (CI), 0.144–0.874; *P* = 0.024), and age (HR, 1.324; 95% CI, 1.063–1.649; *P* = 0.012) and clinical stage (HR, 1.427; 95% CI, 1.138–1.790; *P* = 0.002) were independent risk factors for late LRFFS. For DFFS, nodal stage was an independent unfavourable risk factor (HR, 2.160; 95% CI, 1.646–2.833; *P* < 0.002). Sex, nodal stage and clinical stage were independently associated with EDFFS and LDFFS (all *P* < 0.02). EBV DNA > 4000 copies/ml was independently associated with an increased likelihood of EDFFS (HR, 2.152; 95% CI, 1.615–2.867; *P* < 0.001) but not LDFFS.

**Table 4:** Multivariable analysis of prognostic factors for the entire cohort

End point	Variable	HR	95%CI	*P*-value
**OS**	**Sex(Male vs. Female)**	0.722	0.592–0.882	0.001
	**WHO Histology (I ~ II vs. III)**	0.643	0.443–0.935	0.021
	**Age, year (××45 vs. ≤45)**	1.400	1.193–1.642	< 0.001
	** N stage**^**a**^**( N2-3 vs. N0-1)**	1.417	1.199–1.674	< 0.001
	**Overall stage**^**a**^**( IVa vs. III)**	1.694	1.430–2.007	< 0.001
	**EBV DNA load, copy/ml (≥ 4000 vs. <4000)**	1.462	1.228–1.741	< 0.001
	**CCD, mg/m** ^**2**^			
	0	Reference		
	1-200	0.728	0.609–0.872	0.001
	> 200	0.415	0.263–0.6550	< 0.001
**DFS**	**Sex(Male vs. Female)**	0.713	0.605–0.840	< 0.001
	**WHO Histology (I ~ II vs. III)**	0.627	0.454–0.865	0.004
	**Age, year (××45vs. ≤45)**	1.195	1.046–1.363	0.008
	** N stage**^**a**^**( N2-3 vs. N0-1)**	1.297	1.131–1.488	< 0.001
	**Overall stage**^**a**^**( IVa vs. III)**	1.535	1.336–1.765	< 0.001
	**EBV DNA, copy/ml (≥ 4000 vs. <4000)**	1.387	1.203–1.599	< 0.001
	**CCD, mg/m** ^**2**^			
	0	Reference		
	1-200	0.778	0.668–0.906	0.001
	> 200	0.531	0.376–0.750	< 0.001
**LRFFS**	**WHO Histology (I ~ II vs. III)**	0.610	0.380–0.979	0.040
	**Overall stage**^**a**^**( IVa vs. III)**	1.449	1.185–1.773	< 0.001
**ELRRFS**	**WHO Histology (I ~ II vs. III)**	0.354	0.144–0.874	0.024
**LLRRFS**	**Age, year (××45 vs. ≤45)**	1.324	1.063–1.649	0.012
	**Overall stage**^**a**^**( IVa vs. III)**	1.427	1.138–1.790	0.002
**DMFS**	**N stage**^**a**^**(N0-1 vs.N2-3)**	4.037	2.207–7.383	< 0.001
**EDMFS**	**Sex(Male vs. Female)**	0.674	0.497–0.913	0.011
	** N stage**^**a**^**( N2-3 vs. N0-1)**	2.160	1.646–2.833	< 0.001
	**Overall stage**^**a**^**( IVa vs. III)**	1.376	1.070–1.770	0.013
	**EBV DNA, copy/ml (≥ 4000 vs. <4000)**	2.152	1.615–2.867	< 0.001
	**CCD, mg/m** ^**2**^			
	0	Reference		
	1-200	0.763	0.581–1.002	0.052
	> 200	0.351	0.169–0.732	0.005
**LDMFS**	**Sex(Male vs. Female)**	0.667	0.480–0.926	0.016
	**WHO Histology (I ~ II vs. III)**	0.481	0.275–0.843	0.011
	** N stage**^**a**^**( N2-3 vs. N0-1)**	1.549	1.184–2.025	0.001
	**Overall stage**^**a**^**( IVa vs. III)**	1.608	1.230–2.102	0.001

A Cox proportional hazards regression model was used to detect variables individually without adjustment. All variables were transformed into categorical variables. HRs were calculated for sex, WHO Histology, age, Family history, T stage, N stage, clinical stage, EBV-DNA before treatment, IC cycle, CCD

*Abbreviations:* *HR* Hazard ratio, *CI* Confidence interval, *OS* Overall survival, *DFS* Disease- free survival, *ELRFFS* Early locoregional failure- free survival, *LLRFFS* Late locoregional failure- free survival, *EDFFS* Early distant failure-free survival, *LDFFS* Late distant failure-free survival, *WHO* World Health Organization, *EBV* Epstein–Barr virus, *CCD* Cumulative cisplatin dose

^a^According to the 8th edition of the American Joint Commission on Cancer staging system

### Cisplatin-based concurrent chemotherapy and the timing of treatment failure

**Fig. 3:**
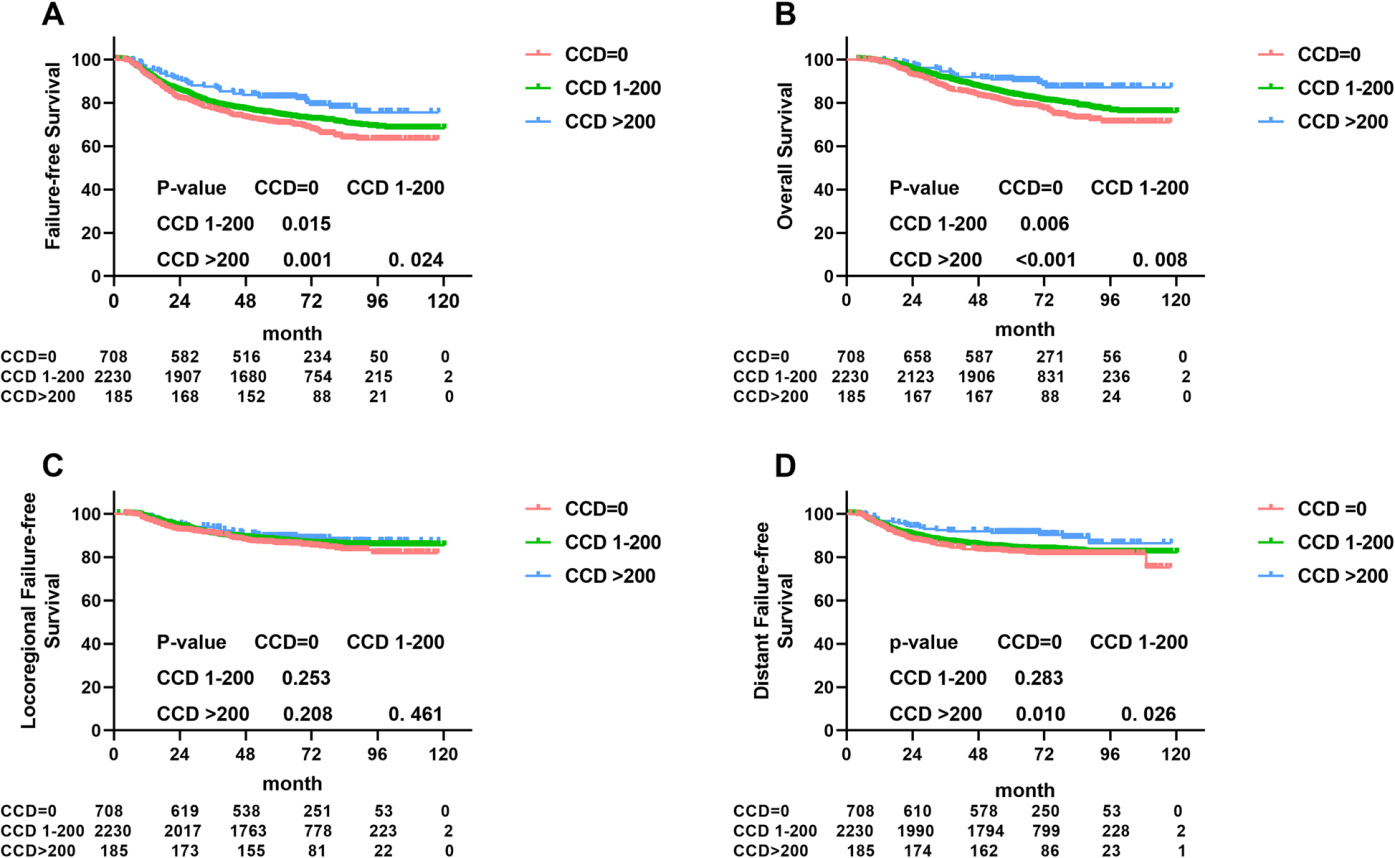
Kaplan–Meier FFS (**A**), OS (**B**), LRFS (**C**) and DMFS (**D**) curves in 3123 LANPC patients stratified by CCD = 0 mg/m2, CCD 1-200 mg/m2, and CCD 200 mg/m2

Kaplan-Meier survival analysis indicated that patients with cisplatin-based concurrent chemotherapy had higher 5-year FFS and OS rates than patients without, and CCD > 200 mg/m^2^ presented a better survival than a medium-level CCD (101–200 mg/m^2^) ([0 mg/m^2^ vs. 1-200 mg/m^2^ vs. >200 mg/m^2^]: FFS: 70.4% vs. 74.4% vs. 82.6%, all *P* < 0.03, Fig. 3A; OS: 79.5% vs. 83.8% vs. 90.8%, all *P* < 0.01, Fig. 3B).

Although there was no significant difference between patients without concurrent cisplatin who received a medium dose in terms of 5-year DFFS (82.2% vs. 84.1%, *P* = 0.283, Fig. 3D), CCD > 200 mg/m^2^ significantly improved DFFS compared to the other groups ([> 200 mg/m^2^ vs. 0 mg/m^2^]: 91.3% vs. 82.2%, *P* = 0.010; [> 200 mg/m^2^ vs. 1-200 mg/m^2^]: 91.3% vs. 84.1%, *P* = 0.026, Fig. 3D). However, no significantly different survival outcomes were observed in different applications of CCD regarding LRFFS ([0 mg/m^2^ vs. 1-200 mg/m^2^ vs. >200 mg/m^2^]: 86.1% vs. 87.6% vs. 89.4%, all P > 0.05, Fig. 3C).

Cox regression modelling predicted that cisplatin-based concurrent chemotherapy was an independent positive factor for OS, FFS and EDFFS (all *P* < 0.05, Table 2). CCD > 200 g/m^2^ was a significant factor in reducing the risk of early distant failure (HR, 0.351; 95% CI, 0.169–0.732; *P* = 0.005, Table 2).

**Fig. 4:**
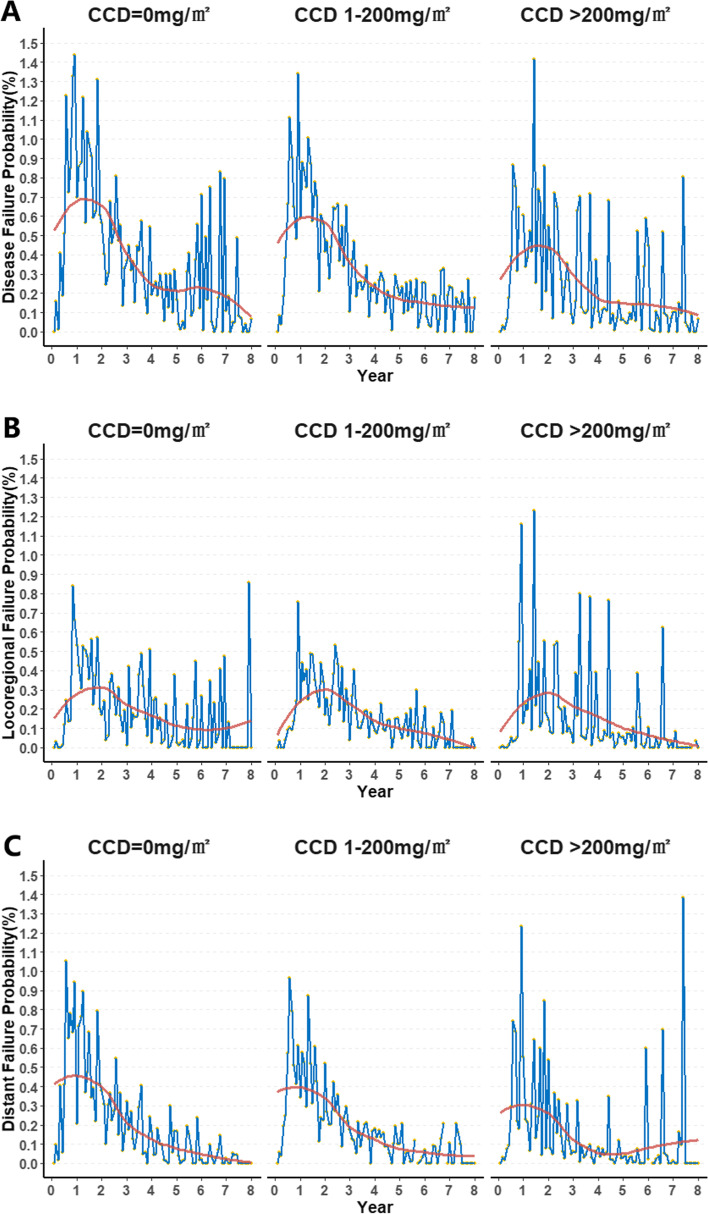
Time-specific treatment failure probabilities (**A**), locoregional failure (**B**), and distant failure probabilities (**C**) in 3123 nasopharyngeal carcinoma patients stratified by CCD = 0 mg/m2, CCD 1-200 mg/m2, and CCD 200 mg/m2

Cumulative risk is summarized in the Supplementary files 2. The monthly risk-adjusted event-occurring probability for the three groups is summarized in Fig. 4. Generally, disease failure, including locoregional recurrence and distant metastasis, mainly occurred in the first 3 years (Fig. 4A, B, C). The peak occurrence time for all endpoints was approximate in all groups, but the lowest probability of distant failure and disease failure was observed in the CCD > 200 g/m^2^ group (Fig. 4A, C). It is worth noting that there was still a small rise in distant metastasis in patients who received concurrent cisplatin after 5 years of follow-up (Fig. 4C). For locoregional recurrence, the probability at peak was approximate, but locoregional recurrences slightly increased in patients without concurrent cisplatin after the 7th year of follow-up (Fig. 4B).

## Discussion

Presently, there is no established and evidence-based definition for early treatment failure of LANPC after radical radiotherapy. With the primary goal of classifying patients into early and late treatment failure groups, we performed a real-world study based on the statistical assessment of the best cut-off value to differentiate prognosis. In our data, early locoregional failure should be defined as recurrence within 14 months, and early distant failure should be defined as recurrence within 20 months. Since IC + CCRT was recommended for LANPC in the latest guidelines, we also investigated the prognostic effect of concurrent chemotherapy and found that cisplatin administered during radiotherapy yielded a survival benefit mainly by reducing the risk of early distant failure.

Throughout the present literature, early treatment failure is related to lower OS in a variety of malignancies [16, 18, 27, 28]. However, the use of OS as the outcome indicator may be biased in determining early and late failure because patients who develop late treatment failure experience a long period without events, and thus, their OS is expected to be longer. According to previous studies, Li and his colleagues defined early local, regional or locoregional recurrence within 2 years for NPC and found that patients with early recurrence demonstrated significantly better OS than patients with late recurrence, but post-recurrence OS did not reach significance[16]. In the present study, post-failure OS was used as the primary outcome, and early failure was defined as a locoregional failure-free interval of 14 months and a distant failure-free interval of 20 months after radiotherapy. Patients who developed early disease failure suffered poorer post-failure OS than those who developed late failure, which should be ascribed to individualized hyposensitivity to chemoradiotherapy and insufficient primary treatment.

Multivariate Cox regression analysis revealed that clinical factors, which correlative with tumor intrinsic biology and dissemination, such as histology type, tumor stage and pretreatment EBV DNA level, were independent factors for early treatment failure. In a study conducted by Groot and his colleagues, preoperatively (> 210U/mL) and postoperatively (> 37U/mL) elevated CA 19–9 were shown to be independent factors for early recurrence in resected pancreatic ductal adenocarcinoma [19]. In intermediate-risk Papillary Thyroid Cancer, significant association was found between Pre-ablation Tg levels > 10 ng/ml and “early” recurrences by Olier et al. [17]. Our study suggested increased pretreatment EBV DNA load was relative with OS and early distant failure, but not late failure. Theoretically, EBV DNA could be released upon cancer cell apoptosis or necrosis [29–31]. The role of pretreatment EBV DNA load for clinical prognostication has been determined in recent years [32, 33], which suggests that this biomarker contain crucial additional biological information regarding tumor intrinsic aggressiveness.

Tumour biology, especially treatment sensitivity, plays an important role in failure time. Speers et al. identified 485 genes that were significantly associated with recurrence time (early vs. late) for women with breast cancer treated with radiation [18]. They also found an association between early recurrence with proliferation and EGFR concepts and late recurrence with luminal and ER signalling pathways. Our data indicated that undifferentiated squamous cell carcinoma (WHO type III) was less likely to suffer early locoregional recurrence. One of probable hypothesis is the discrepancy of NPC tumor cell radiosensitivity. Recent studies have shown that apoptosis, DNA damage repair, a hypoxic microenvironment, and autophagy can be involved in regulating radiotherapy resistance [34–36]. Moreover, post failure treatment was a significant prognosis factor for post failure survival, in which supportive treatment was associated with poorer post-recurrence OS compared with salvage surgery, re-irradiation and chemotherapy [16].

Treatment strategies also influence disease failure, and thus, more attention should be paid to the initial treatment. In patients with gastric cancer after radical gastrectomy, > 3 cycles of postoperative adjuvant chemotherapy (AC) significantly reduced the risk of early recurrence in the pathological stage III and high CAR subgroups (16.0% vs. 54.2%, *p* = 0.004) [20]. The observations by Sargent and his colleagues, based on 18 randomized trials of patients with stage II or III colon cancer, demonstrated that AC significantly and meaningfully improved long-term survival by reducing the risk of early recurrence [37]. In the present study, concurrent chemotherapy provided a significant FFS benefit for locoregionally advanced NPC primarily by reducing the distant failure rate. Monthly analysis demonstrated that the risk of failure in each group was dominated by the early period, particularly the first 3 years. Concurrent cisplatin administration mainly decreased the magnitude of the peak in the early period of follow-up, especially reducing the risk of distant metastasis. It is well documented that distant metastasis has become the main cause of treatment failure for NPC patients [5–7]. Since EDM was related to lower survival, early DFFS improvement could translate into a longer-term OS benefit.

The interaction of radiation and cisplatin is likely to enhance cytotoxicity and thus improve locoregional control [38], but it seems that a low CCD was not sufficient to reduce the risk of locoregional relapse. In a dose-effect analysis conducted by Peng et al., a CCD of 230–270 mg/m^2^ played an important role in improving LRFS in patients who received CCRT alone [39]. In the combined analyses of NPC-9901 and NPC-9902, a total dose of cisplatin during the concurrent phase (> 200 mg/m^2^) yielded a significant benefit on the LRFS and OS stage III subgroups [8]. For patients who received IC + CCRT, a high CCD yielded more beneficial antitumour effects on OS, FFS, and DFFS but not LRFFS [12, 40], which is consistent with our findings. This discrepancy may be caused by the varied population demographics, inclusion criteria and follow-up times in each study. Another possible explanation is the confounding effects of IC, even after the multivariate analysis was conducted.

CCD ≥ 200 mg/m ^2^ may be indicated for high-risk patients with unfavorable response to IC. Liu et al. found that NPC patients receiving complete tumor response (CR)/partial response (PR) after IC benefited from ≥ 200 mg/m ^2^ CCD during CCRT; while those with stable disease (SD)/disease progression (PD) didn’t. They indicated that patients with good response to IC might be inappropriate to reduce concurrent CCD because they may benefit from higher CCD. Notably, concurrent cisplatin treatment actually eradicated micrometastasis foci and postponed failure in our study. The incidence of distant metastasis climbed in the high CCD group after 5 years of follow-up, which might be due to the cessation of inhibition by chemotherapeutic agents. In such cases, longer-term follow-up is required to verify a long-term survival benefit, as effective therapy has extended survival regardless of the agent’s mechanism.

This is the first study to establish an evidence-based cut-off value of disease failure for LANPC. Further investigation of the failure pattern with or without concurrent cisplatin is relevant to our understanding of tumour biology, clinical practice, and future clinical trial design. Nevertheless, there were several limitations in this study due to the retrospective nature. First, bias was inevitable as patient selection and treatment decisions were made, especially the heterogeneity of the IC and the treatment after failure. Second, toxicity and potential confounders, including patient compliance and social and economic factors, were unavailable in our data. Last, the limited numbers in some subsets may weaken the statistical power of the analyses. Further prospective studies are required to confirm the preliminary result of this study.

## Conclusion

In summary, there is presently no established and evidence-based threshold to differentiate between early and late failure following radical radiotherapy for LANPC. This study found locoregional failure within 14 months and distant failure within 20 months to be the optimal definition of early disease failure based on subsequent prognosis. Furthermore, in patients who receive cisplatin during radiotherapy, the risk of recurrence is dominated by the early follow-up period, particularly the first 3 years. The administration of concurrent cisplatin significantly and meaningfully reduced the risk of early distant failure and thus improved OS.

## Supplementary Information

**Additional file 1:** R code for minimum *P*-value approach.

**Additional file 2:** **Supplementary files 2.** Cumulative risk of treatment failure (A), locoregional failure (B), and distant failure probabilities (C) in 3123 nasopharyngeal carcinoma patients stratified by CCD = 0 mgm2, CCD 1-200 mgm2, and CCD 200 mg/m2.

**Additional file 3:** **Supplementary table1.** Demographics and clinical characteristics of patients with failure.

## Data Availability

The authenticity of this article has been validated by uploading the key raw data onto the Research Data Deposit (RDD) public platform (http://www.researchdata.org.cn), with the RDD approval number of RDDA2021001983. The data that support the findings of this study are available from RDD, but restrictions apply to the availability of these data, which were used under license for the current study, and so are not publicly available. Data are however available from the authors upon reasonable request and with permission of RDD.

## Abbreviations

LANPC: Locoregionally advanced nasopharyngeal carcinoma
RSF: Random survival forest
ELRF: Early locoregional failure
LLRF: Late locoregional failure
EDF: Early distant failure
LDF: Late distant failure
HR: Hazard ratio
CI: Confidence interval
FFS: Failure-free survival
OS: Overall survival
IC: Induction chemotherapy
CCRT: Concurrent chemoradiotherapy
NCCN: National Comprehensive Cancer Network
CCD: Cumulative cisplatin dose
SYSUCC: Sun Yat-sen University Cancer Center
AJCC: American Joint Committee on Cancer
UICC: International Union against Cancer
IMRT: Intensity-modulated radiation therapy
DFFS: Distant failure-free survival
LRFFS: Locoregional failure-free survival
