# Supplementary material for: Cisplatin-based concurrent chemoradiotherapy improved the survival of locoregionally advanced nasopharyngeal carcinoma after induction chemotherapy by reducing early treatment failure
Source: BMC Cancer. 2022 Nov 29;22:1230. doi: 10.1186/s12885-022-10237-8 (PMC9706941; doi:10.1186/s12885-022-10237-8)
Supplement: Supplementary file 1 — Additional file 1: R code for minimum P-value approach. [file 12885_2022_10237_MOESM1_ESM.docx]

# Run the function first

MinmumPValue <- function(TimeUse, StateUse, VarUse, Btime){

# Calculate the maximum chi-squared value

VarUniSort <- unique(sort(VarUse)) # Sort independent variables from smallest to largest and eliminate duplicate values

NumPeo <- length(VarUse) # Extract the total number of rows (i.e., the total number of people)

NumVarUni <- length(VarUniSort) # Extract the number of independent variable values that are not repeated

ChisqList <- rep(0,NumVarUni-1) # Initializes the result list ，save the chi-square value for each partition

for (i in 1:(NumVarUni-1)) { # star running

VarTwoStage <- rep(0,NumPeo) # Create variable VarTwoStage with value 1 if greater than the threshold and 0 otherwise

VarTwoStage[VarUse>VarUniSort[i]] <- 1

KMSummary <- survdiff(Surv(TimeUse, StateUse) ~ VarTwoStage) # The log-rank test calculates the chi-squared value

ChisqList[i] <- KMSummary$chisq # Save the chi-square value to the ChiSqList

}

ChisqMax <- max(ChisqList) # Extract maximum chi-square value

POri <- 1 - pchisq(ChisqMax,1) # Calculate P value (uncorrected P value)

VarSuit <- VarUniSort[which(ChisqList==max(ChisqList))] # Extract the threshold（≤ & >）

OutComeDF <- data.frame(var = VarUniSort[1:(NumVarUni-1)],

Chisq = ChisqList,

P = 1 - pchisq(ChisqList,1))# P value was adjusted by approximate method, reference：DOI:10.2307/2529881

z <- qnorm(1 - POri/2)

e <- c(0.15, 0.10, 0.05, 0.01, 0.001, 1/NumPeo)

PAdjAppr <- dnorm(z)*(z-1/z)*log( ((1-e)^2) / (e^2) ) + 4*dnorm(z)/z # P value (approximate method)

# adjust P value with Bootstrap，book：Bootstrap Methods:A Guide for Practioners and Researchers, 2nd Edition

ChisqListB <- rep(0, Btime)

for (iB in 1:Btime) {

IDUse <- sample(1:NumPeo,NumPeo,replace = TRUE)

TimeUseB <- TimeUse[IDUse]

StateUseB <- StateUse[IDUse]

VarUseB <- VarUse[IDUse]

VarUniSortB <- unique(sort(VarUse[IDUse]))

NumPeoB <- length(VarUse[IDUse])

NumVarUniB <- length(VarUniSortB)

ChisqList <- rep(0,NumVarUniB-1)

for (i in 1:(NumVarUniB-1)) {

VarTwoStage <- rep(0,NumPeoB)

VarTwoStage[VarUseB>VarUniSortB[i]] <- 1

KMSummary <- survdiff(Surv(TimeUseB, StateUseB) ~ VarTwoStage)

ChisqList[i] <- KMSummary$chisq

}

ChisqListB[iB] <- max(ChisqList)

}

PAdjBoot <- sum(ChisqListB >= ChisqMax) / Btime # P value Bootstrap法

OutcomeText <- paste0("The optimal grouping is：<=",VarSuit,"as group1，>",VarSuit,

"as group2;\the maximum chi-squared value",round(ChisqMax,3),"\n",

"un adjusted P value: ",POri,"\n",

"P value adjusted by approximate method:\n",

" ε=0.15: ",PAdjAppr[1],"\n",

" ε=0.10: ",PAdjAppr[2],"\n",

" ε=0.05: ",PAdjAppr[3],"\n",

" ε=0.01: ",PAdjAppr[4],"\n",

" ε=0.001: ",PAdjAppr[5],"\n",

" ε=",round(1/NumPeo,5),": ",PAdjAppr[6],

"P value was adjusted by Bootstrap(B=",Btime,"): ",PAdjBoot)

cat(OutcomeText)

return(OutComeDF)

}

################################################################################################################################################

data<-read.table

library("survival")

TimeUse <- ovarian$futime # survial time

StateUse <- ovarian$fustat # survival outcome（1=death，0= Censored）

VarUse <- ovarian$age # The independent variable to be discretized

Btime <- 10000

# Repeat times of Bootstrap method,More than 5,000 is recommended

VarList <- MinmumPValue(data$futime, data$fustat, data$age, Btime)
