# Supplementary material for: Cisplatin-based concurrent chemoradiotherapy improved the survival of locoregionally advanced nasopharyngeal carcinoma after induction chemotherapy by reducing early treatment failure
Source: BMC Cancer. 2022 Nov 29;22:1230. doi: 10.1186/s12885-022-10237-8 (PMC9706941; doi:10.1186/s12885-022-10237-8)
Supplement: Supplementary file 2 — Additional file 2: Supplementary files 2. Cumulative risk of treatment failure (A), locoregional failure (B), and distant failure probabilities (C) in 3123 nasopharyngeal carcinoma patients stratified by CCD = 0 mgm2, CCD 1-200 mgm2, and CCD 200 mg/m2. [file 12885_2022_10237_MOESM2_ESM.docx]

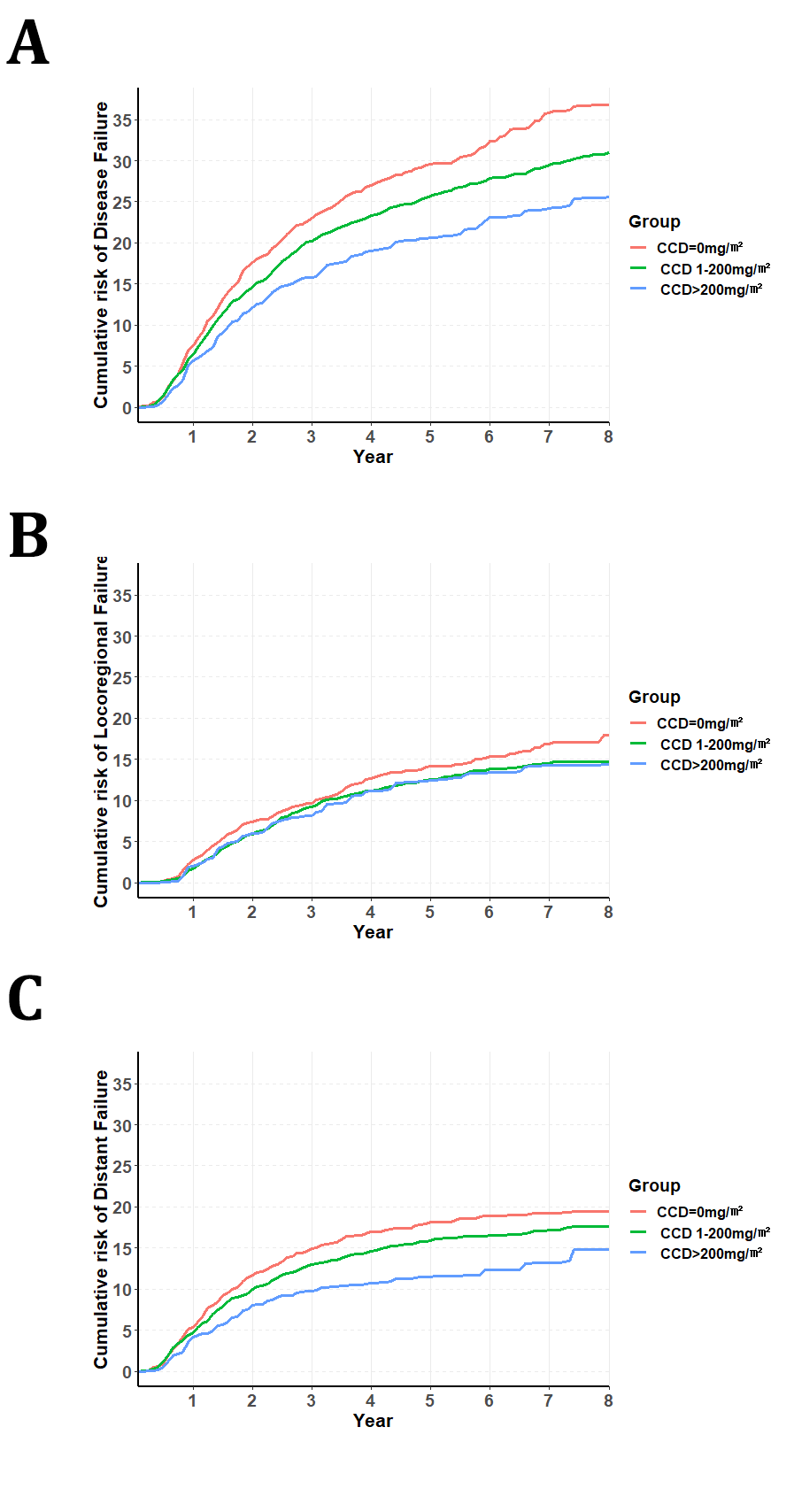


**Supplementary files 2.** Cumulative risk of treatment failure (A), locoregional failure (B), and distant failure probabilities (C) in 3123 nasopharyngeal carcinoma patients stratified by CCD = 0 mg/m^2^, CCD 1-200 mg/m^2^, and CCD>200 m^2^. CCD, cumulative cisplatin dose
