# Supplementary material for: Cisplatin-based concurrent chemoradiotherapy improved the survival of locoregionally advanced nasopharyngeal carcinoma after induction chemotherapy by reducing early treatment failure
Source: BMC Cancer. 2022 Nov 29;22:1230. doi: 10.1186/s12885-022-10237-8 (PMC9706941; doi:10.1186/s12885-022-10237-8)
Supplement: Supplementary file 3 — Additional file 3: Supplementary table1. Demographics and clinical characteristics of patients with failure. [file 12885_2022_10237_MOESM3_ESM.docx]

Supplementary table 1. Demographics and clinical characteristics of patients with failure.

| **Characteristic** | **No. of patients*** | | | **P value** | **No. of patients*** | | | **P value** |
| --- | --- | --- | --- | --- | --- | --- | --- | --- |
|  | **ELRF** | **LLRF** | **Total** |  | **EDF** | **LDF** | **Total** |  |
|  | 88(100.0%) | 319(100.0%) | 407(100.0%) |  | 278(100.0%) | 233(100.0%) | 511(100.0%) |  |
| **Sex** |  |  |  | 0.774 |  |  |  | 0.909 |
| Male | 67(76.1%) | 248(77.7%) | 315(77.4%) |  | 227(81.7%) | 189(81.1%) | 416(81.4%) |  |
| Female | 21(23.9%) | 71(22.3%) | 92(22.6%) |  | 51(18.3%) | 44(18.9%) | 95(18.6%) |  |
| **Histology** |  |  |  | 0.241 |  |  |  | 0.409 |
| WHO Type I-II | 6(6.8%) | 12(3.8%) | 18(4.4%) |  | 11(4.0%) | 13(5.6%) | 24(4.7%) |  |
| WHO Type III | 82(93.2%) | 307(96.2%) | 389(95.6%) |  | 267(96.0%) | 220(94.4%) | 487(95.3%) |  |
| **Age, year** |  |  |  | 0.016 |  |  |  | 0.013 |
| ≤45 | 33(37.5%) | 166(52.0%) | 199(48.9%) |  | 121(43.5%) | 128(54.9%) | 249(48.7%) |  |
| ＞45 | 55(62.5%) | 153(48.0%) | 208(51.1%) |  | 157(56.5%) | 105(45.1%) | 262(51.3%) |  |
| **Smoking history** |  |  |  | 0.389 |  |  |  | 0.858 |
| No | 50(56.8%) | 198(62.1%) | 248(60.9%) |  | 161(57.9%) | 133(57.1%) | 294(57.5%) |  |
| Yes | 38(43.2%) | 121(37.9%) | 159(39.1%) |  | 117(42.1%) | 100(42.9%) | 217(42.5%) |  |
| **Drinking history** |  |  |  | 0.470 |  |  |  | 0.910 |
| No | 75(85.2%) | 281(88.1%) | 356(87.5%) |  | 225(80.9%) | 190(81.5%) | 415(81.2%) |  |
| Yes | 13(14.8%) | 38(11.9%) | 51(12.5%) |  | 53(19.1%) | 43(18.5%) | 96(18.8%) |  |
| **Family history of cancer** |  |  |  | 0.786 |  |  |  | 0.114 |
| No | 66(75%) | 234(73.4%) | 300(73.3%) |  | 208(74.8%) | 159(68.2%) | 367(71.8%) |  |
| Yes | 22(25.0%) | 85(26.6%) | 107(26.3%) |  | 70(25.2%) | 74(31.8%) | 144(28.2%) |  |
| **T stage**** |  |  |  | 0.695 |  |  |  | 0.142 |
| T1-2 | 10(11.4%) | 32(10.0%) | 42(10.3%) |  | 50(18.0%) | 30(12.9%) | 80(15.7%) |  |
| T3-4 | 78(88.6%) | 287(90.0%) | 365(89.7%) |  | 228(82.0%) | 203(87.1%) | 431(84.3%) |  |
| N stage** |  |  |  | 0.149 |  |  |  | 0.006 |
| N0-1 | 36(40.9%) | 159(49.8%) | 195(47.9%) |  | 74(26.6%) | 89(38.2%) | 163(31.9%) |  |
| N2-3 | 52(59.1%) | 160(50.2%) | 212(52.1%) |  | 204(73.4%) | 144(61.8%) | 348(68.1%) |  |
| **Overall stage**** |  |  |  | 0.714 |  |  |  | 0.713 |
| III | 34(38.6%) | 132(41.4%) | 166(40.8%) |  | 101(36.3% | 89(38.2%) | 190(37.2%) |  |
| IVa | 54(61.4%) | 187(58.6%) | 241(59.2%) |  | 177(63.7%) | 144(61.8%) | 321(62.8%) |  |
| **EBV DNA load, copy/ml** |  |  |  | 1.000 |  |  |  | <0.002 |
| <4000 | 33(37.5%) | 120(37.6%) | 153(37.6%) |  | 63(22.7%) | 86(36.9%) | 149(29.2%) |  |
| ≥4000 | 55(62.5%) | 199(62.4%) | 254(62.4%) |  | 215(77.3%) | 147(63.1%) | 362(70.8%) |  |
| **IC cycles** |  |  |  | 0.631 |  |  |  | 0.010 |
| ≤2 cycles | 43(48.9%) | 166(52.0%) | 209(51.4%) |  | 134(48.2%) | 139(59.7%) | 273(53.4%) |  |
| >2 cycles | 45(51.1%) | 153(48.0%) | 198(48.6%) |  | 144(51.8%) | 94(40.3%) | 238(46.6%) |  |
| **Salvage treatment** |  |  |  | 0.064 |  |  |  | 0.016 |
| Supportive treatment | 8(9.1%) | 24(7.5%) | 32(7.9%) |  | 63(22.7%) | 51(21.9%) | 114(22.3%) |  |
| Surgery | 22(25.0%) | 65(20.4%) | 87(21.4%) |  | 24(8.6%) | 30(12.9%) | 54(10.6%) |  |
| Radiotherapy | 18(20.0%) | 100(831.3%) | 118(29.0%) |  | 15(5.4%) | 19(8.2%) | 34(6.7%) |  |
| Chemotherapy | 40(45.5%) | 119(37.3%) | 159(39.1%) |  | 161(57.9%) | 112(48.1%) | 273(53.4%) |  |
| Comprehensive therapy*** | 0(0%) | 6(1.9%) | 6(1.5%) |  | 14(5.0%) | 12(5.2%) | 26(5.1%) |  |
| unknown | 0(0%) | 5(1.6%) | 5(1.2%) |  | 1(0.4%) | 9(3.9%) | 10(2.0%) |  |

Abbreviations: ELRF = early locoregional failure; LLRF = late locoregional failure; EDF = early distant failure; LDF= late distant failure; WHO = World Health Organization; EBV = Epstein–Barr virus.
* Percentages may not add up to 100 due to rounding.

**According to the 8th edition of the American Joint Commission on Cancer staging system.

***patient received more than one kind of salvage treatment (Surgery, Radiotherapy, Chemotherapy).
